# A High Rate of Recurrent Vulvovaginal Candidiasis and Therapeutic Failure of Azole Derivatives Among Iranian Women

**DOI:** 10.3389/fmicb.2021.655069

**Published:** 2021-04-28

**Authors:** Amir Arastehfar, Melika Laal Kargar, Shahla Roudbar Mohammadi, Maryam Roudbary, Nayereh Ghods, Ladan Haghighi, Farnaz Daneshnia, Mahin Tavakoli, Jalal Jafarzadeh, Mohammad Taghi Hedayati, Huiwei Wang, Wenjie Fang, Agostinho Carvalho, Macit Ilkit, David S. Perlin, Cornelia Lass-Flörl

**Affiliations:** ^1^Center for Discovery and Innovation, Hackensack Meridian Health, Nutley, NJ, United States; ^2^Department of Mycology, Faculty of Medical Science, Tarbiat Modares University, Tehran, Iran; ^3^Department of Parasitology and Mycology, School of Medicine, Iran University of Medical Sciences, Tehran, Iran; ^4^Department of Obstetrics and Gynecology, School of Medicine, Iran University of Medical Sciences, Tehran, Iran; ^5^Department of Medical Mycology and Parasitology, School of Medicine, Babol University of Medical Sciences, Babol, Iran; ^6^Invasive Fungi Research Center, Department of Medical Mycology, School of Medicine, Mazandaran University of Medical Sciences, Sari, Iran; ^7^Shanghai Key Laboratory of Molecular Medical Mycology, Shanghai Institute of Mycology, Shanghai Changzheng Hospital, Second Military Medical University, Shanghai, China; ^8^Department of Dermatology, Shanghai Changzheng Hospital, Second Military Medical University, Shanghai, China; ^9^Life and Health Sciences Research Institute (ICVS), School of Medicine, University of Minho, Braga, Portugal; ^10^ICVS/3B’s – PT Government Associate Laboratory, Guimarães/Braga, Portugal; ^11^Division of Mycology, University of Çukurova, Adana, Turkey; ^12^Division of Hygiene and Medical Microbiology, Medical University of Innsbruck, Innsbruck, Austria

**Keywords:** recurrent vulvovaginal candidiasis, *Candida albicans*, *Candida glabrata*, fluconazole therapeutic failure, fluconazole tolerance, fluconazole resistance

## Abstract

Recurrent vulvovaginal candidiasis (RVVC) is one of the most prevalent fungal infections in humans, especially in developing countries; however, it is underestimated and regarded as an easy-to-treat condition. RVVC may be caused by dysbiosis of the microbiome and other host-, pathogen-, and antifungal drug-related factors. Although multiple studies on host-related factors affecting the outcome have been conducted, such studies on *Candida*-derived factors and their association with RVVC are lacking. Thus, fluconazole-tolerant (FLZT) isolates may cause fluconazole therapeutic failure (FTF), but this concept has not been assessed in the context of *Candida*-associated vaginitis. Iran is among the countries with the highest burden of RVVC; however, comprehensive studies detailing the clinical and microbiological features of this complication are scarce. Therefore, we conducted a 1-year prospective study with the aim to determine the RVVC burden among women referred to a gynecology hospital in Tehran, the association of the previous exposure to clotrimazole and fluconazole with the emergence of FLZT and fluconazole-resistant (FLZR) *Candida* isolates, and the relevance of these phenotypes to FTF. The results indicated that about 53% of the patients (43/81) experienced RVVC. *Candida albicans* and *C*. *glabrata* constituted approximately 90% of the yeast isolates (72 patients). Except for one FLZT *C*. *tropicalis* isolate, FLZR and FLZT phenotypes were detected exclusively in patients with RVVC; among them, 27.9% (12/43) harbored FLZR strains. *C. albicans* constituted 81.2% of FLZR (13/16) and 100% of the FLZT (13/13) isolates, respectively, and both phenotypes were likely responsible for FTF, which was also observed among patients with RVVC infected with fluconazole-susceptible isolates. Thus, FTF could be due to host-, drug-, and pathogen-related characteristics. Our study indicates that FLZT and FLZR isolates may arise following the exposure to over-the-counter (OTC) topical azole (clotrimazole) and that both phenotypes can cause FTF. Therefore, the widespread use of OTC azoles can influence fluconazole therapeutic success, highlighting the necessity of controlling the use of weak topical antifungals among Iranian women.

## Introduction

Fungi are major components of the human microbiome (Rolling et al., 2020) and are associated with approximately 1.7 billion superficial fungal infections (SFIs) and 1.5 million deaths due to invasive fungal infections (IFIs) (Brown et al., 2012). While IFIs have received notable attention of medical mycologists (Brown et al., 2012), SFIs are somewhat neglected, being considered as mild and easily treatable conditions. Vulvovaginal candidiasis (VVC) is one of the most prevalent manifestations of SFIs, and it is estimated that 75% of women experience at least one VVC episode during their lifetime (Brown et al., 2012; Denning et al., 2018). Furthermore, approximately 138 million women suffer from recurrent vulvovaginal candidiasis (RVVC) annually, and this number is projected to reach 158 million by 2030 (Denning et al., 2018). RVVC can severely affect the quality of life for the afflicted patients and imposes a significant economic burden, which exceeds 14.39 billion USD in developed countries (Denning et al., 2018).

Antibiotic overuse, diabetes, pregnancy, and cystic fibrosis are among the risk factors for RVVC (Denning et al., 2018). In addition, immunodeficiency due to genetic aberrations (Jaeger et al., 2016; Tian et al., 2017), local immune overreaction (Rosati et al., 2020), the inefficiency of prescribed antifungal agents, and, to a lesser extent, the development of antifungal resistance contribute to RVVC. Numerous studies have evaluated the association between host-related factors and RVVC (Rosati et al., 2020); however, there is a lack of similar studies on *Candida*-related factors such as drug resistance and tolerance, which limits our understanding of their relevance to RVVC. Recently, it has been reported that tolerance to the principal antifungal agent fluconazole among a subpopulation of susceptible isolates may promote colonization, thus increasing therapeutic failure and mortality rates (Astvad et al., 2018; Rosenberg et al., 2018). Fluconazole resistance phenotype is due to stable genomic changes with visible growth of higher than minimum inhibitory concentration (MIC) at 24 h endpoint in the presence of drug. Fluconazole tolerance, on the other hand, is mainly due to physiologic changes allowing a subpopulation of cells, called tolerant cells, to grow slowly at concentrations above MIC for which the data MIC are recorded at 48 h (Rosenberg et al., 2018; Arastehfar et al., 2020c). Therefore, fluconazole tolerance may not be detected using standard broth microdilution assays in which the scoring endpoint is 24 h, indicating the necessity of alternative analytical methods (Rosenberg et al., 2018). Although drug tolerance has been studied in the context of candidemia, its role in VVC remains unclear.

Treatment of fungal infections depends on clinical manifestations, disease severity, and causative yeast/*Candida* species (Pappas et al., 2016). A single 150 mg dose of fluconazole is recommended for treating VVC, whereas 10–14-day induction with topical fluconazole or other agents, followed by 150 mg oral fluconazole weekly for a period of 6 months is recommended for RVVC (Pappas et al., 2016). Since some *Candida* species such as *Candida glabrata* can rapidly develop resistance to azoles (Arastehfar et al., 2020c), therefore the treatment of VVC consists of topical intravaginal nystatin or boric acid or 17% flucytosine cream alone or in combination with 3% amphotericin B cream for 14 days (Pappas et al., 2016). Although *Candida albicans* has been historically known as the most prevalent causative agent of fungal vulvovaginitis, new lines of evidence reveal an increasing incidence of non-*albicans Candida* (NAC) species, which generally respond to higher MICs of azoles (Makanjuola et al., 2018) and which may contribute to complications associated with RVVC. Since the vast majority of RVVC cases are recorded in developing countries (Denning et al., 2018), the shift toward NAC species could be problematic for their healthcare institutions, where the diagnosis and treatment of fungal infections are inadequate (Arastehfar et al., 2019a,b,c,d; Arastehfar et al., 2020d; Kord et al., 2020; Megri et al., 2020).

Iran, a developing country with a population of 85 million people, has been estimated to be among the countries with the highest RVVC prevalence (>4,300 cases per 100,000 women) (Denning et al., 2018); however, there is a lack of comprehensive epidemiological studies detailing the clinical and microbiological characteristics of VVC and RVVC. Therefore, the aim of the current prospective study conducted in Tehran was to determine the prevalence of VVC and RVVC and the rate of therapeutic failure for commonly used antifungals. We also assessed the proportion of fluconazole-resistant (FLZR) and fluconazole-tolerant (FLZT) isolates and examined their association with prior azole exposure and azole therapeutic failure. Thus, the novelty of our study was the focus on fungal factors affecting the efficacy of treatment.

## Materials and Methods

### Patients, Definitions, and Treatment Strategies

Women referred to the Shahid Akbar-Abadi Obstetrics and Gynecology Hospital in Tehran, Iran, between January 30, 2018 and January 30, 2019 were included in the current prospective study. Swab samples were obtained from patients with symptoms including but not limited to vulvar pruritus, burning vaginal soreness, dyspareunia and dysuria, edema, fissures, and vulvar and vaginal erythema. VVC was confirmed by microscopic detection of yeast structures and yeast/*Candida*-positive cultures (Gonçalves et al., 2016; Denning et al., 2018). Cases of bacterial vaginosis were excluded from this study.

Antifungal treatment did not depend on culture results and was empirically prescribed by the treating gynecologist based on gynecologic examination and microscopic observations. Patients were treated at the discretion of the treating gynecologists. Initial antifungal treatment included two 150-mg doses of oral fluconazole (the 1st and 4th days) or 1% topical clotrimazole (for 10–12 days). Patients showing remission after clotrimazole treatment were switched to two 150-mg doses of fluconazole, whereas those with remission after receiving oral fluconazole were switched to two doses of fluconazole + clotrimazole. If remission persisted after 3 months, patients were prescribed two 150-mg doses of fluconazole biweekly for 6 months.

Recurrent vulvovaginal candidiasis was diagnosed by treating physicians based on the following criteria: the patient developed ≥3 episodes per year (Rosati et al., 2020), the initial antifungal treatment did not result in improvement, and *Candida* species were detected by both microscopy and culture. Moreover, patients who used over-the-counter (OTC) clotrimazole for a long time (over 1 year) to treat recurrent complications prior to this study were considered as having RVVC. Short-term exposure was defined when patients (had) completed clotrimazole therapy (10–12 days). Follow-up was conducted by the phone every 2 weeks to evaluate the overall improvement and patients with complaints despite treatment were requested to come to the hospital for examination and swab test. Patients with remission who dropped out of the study after completion of the second course of antifungal treatment were monitored by phone calls.

This study was approved by the human subject hospital review board of Shahid Akbar-Abadi Obstetrics and Gynecology Hospital (IR.MODARES.REC.1397.225). Informed consents were obtained from all patients included in the current study, and researchers were blinded to patient identifiers.

### Yeast Isolation and Identification

Vaginal samples were taken from symptomatic patients using sterile cotton swabs and transferred immediately to Falcon tubes containing PBS. Sampling was performed in accordance with institutional safety protocols. First, the specimens were observed directly under a microscope to reveal yeast structures and then cultured on Sabouraud dextrose agar and CHROMagar (Candiselect, Bio-Rad, Hercules, CA, United States) at 35°C for 48 h. DNA was extracted using a acetyl trimethylammonium bromide-based method described previously (Arastehfar et al., 2018). Isolates were primarily identified by a previously developed multiplex PCR, which can identify 21 yeast species associated with human infections (Arastehfar et al., 2019c). This assay includes three multiplex PCR reactions in which the first multiplex PCR identifies the main *Candida* species, the second one identifies the emerging *Candida* species, and the third multiplex PCR identifies basidiomycetous yeast species. Details regarding this assay were presented in details previously and primers used are listed in Supplementary Tables 1–3. Isolates were further identified by MALDI-TOF MS using a full-extraction method (Arastehfar et al., 2019b) to confirm the accuracy of the results obtained by PCR.

### Antifungal Susceptibility Testing

Antifungal susceptibility testing (AFST) was conducted according to the Clinical Laboratory Standards Institute (CLSI-M27) protocol, fourth edition [Clinical and Laboratory Standards Institute (CLSI), 2017]. MIC values were interpreted based on CLSI-M60, second edition [Clinical and Laboratory Standards Institute (CLSI), 2020], previously reported clinical breakpoints (CBPs), and epidemiological cutoff values (ECVs) (Pfaller and Diekema, 2012). AFST included fluconazole and itraconazole (both from Sigma-Aldrich, St. Louis, MO, United States) but not echinocandins and amphotericin B, since patients did not receive these antifungals. Of note, clotrimazole was not included in our AFST scheme due to the lack of CBPs and ECVs to interpret MICs obtained for this antifungal. Antifungal drugs were dissolved in RPMI1640 (Sigma-Aldrich). Plates were incubated at 35°C and MICs were determined after 24 h. *Candida parapsilosis* (ATCC 22019) and *Candida krusei* (ATCC 6258) were used for quality control purposes. The MIC values for species lacking CBPs were interpreted based on ECVs, and isolates showing MICs > ECV or <ECV were considered as non-wild type (NWT) or wild-type (WT), respectively [Pfaller and Diekema, 2012; CLSI, 2nd ed. CLSI supplement M60 Clinical and Laboratory Standards Institute (CLSI), 2017]. Non-susceptible *C*. *albicans*, *C*. *parapsilosis*, and *C*. *tropicalis* isolates were denoted when the MICs were ≥4 μg/ml, while fluconazole-resistant *C*. *glabrata* isolates had MICs ≥ 64 μg/ml. *C. albicans* isolates with itraconazole MICs ≥ 1 μg/ml were considered as resistant and *C*. *glabrata* isolates showing MICs > 2 μg/ml and *C*. *parapsilosis* and *C*. *tropicalis* with MICs > 0.5 μg/ml were regarded as NWT to itraconazole. Since there is no CBPs for *C*. *krusei*, the MIC data were reported per ECVs, where *C*. *krusei* isolates with MICs > 64 μg/ml and >1 μg/ml were noted as NWT to fluconazole and itraconazole (Pfaller and Diekema, 2012).

Isolates growing at concentrations above the MIC after 48 h were considered as FLZT (Arastehfar et al., 2020b; Berman and Krysan, 2020). Fluconazole-tolerance for *C*. *albicans*, *C*. *parapsilosis*, and *C*. *tropicalis* was noted when the 48 h fluconazole MICs were ≥4 μg/ml, while fluconazole tolerance of *C*. *glabrata* was defined when the MICs of 48 h were ≥64 μg/ml (Rosenberg et al., 2018; Arastehfar et al., 2020b).

## Results

### Patients’ Characteristics

During the study period, 300 patients referred to the clinic were screened; 81 had confirmed VVC and over half of them were diagnosed with RVVC (43/81; 53%) (Table 1). The median age for RVVC patients was 35 years (20–68 years) and for patients with VVC only (38/81; 47%), the median age was 39 years (19–63 years). About 88% of patients with VVC and RVVC were healthy; the underlying conditions in the minority of patients included hypothyroidism (4/81, 4.8%; two in each group), diabetes (2/81, 2.4%; one in each group), anemia (2/81, 2.4%; one in each group), fatty liver (1/81, 1.2%; the VVC group), and hypertension (1/81, 1.2%; the VVC group). There was no significant difference in age and the underlying conditions between the VVC and RVVC groups. Among the 43 patients with RVVC, 12 had prior exposure to OTC topical clotrimazole for ≥1 year (Table 2). During the study period, nine and 15 patients with RVVC dropped out of the study after completing the second and third course of treatment, respectively. Approximately 62% of patients with RVVC had persistent vaginal candidiasis (28/43), whereas for all patients with VVC the infection was cleared after the first use of azoles and no signs of infection were recorded through the entire follow-up period.

**TABLE 1:** Isolate and patient numbers and *Candida* species distribution among patients with VVC and RVVC.

Species (number of isolates;%)	VVC number of cases (number of isolate)	RVVC number of cases (number of isolate)	Total number of patients (%)
*Candida albicans* (*n* = 114; 78.6%)	29 (*n* = 29)	35 (*n* = 85)	(64/81; 79%)
*Candida glabrata* (*n* = 17; 11.7%)	3 (*n* = 3)	5 (*n* = 14)	(8/81; 9.9%)
*Candida krusei* (*n* = 11; 7.5%)	3 (*n* = 3)	3 (*n* = 8)	(6/81; 7.4%)
*Candida parapsilosis* (*n* = 2; 1.3%)	2 (*n* = 2)	0	(2/81; 2.4%)
*Candida tropicalis* (*n* = 1; 0.68%)	1 (*n* = 1)	0	(1/81; 1.2%)
(*n* = 145; 100%)	38 (*n* = 38) (38/107; 35.5%)	43 (*n* = 107) (107/145; 73.7%)	(81; 100%)

*VVC, vulvovaginal candidiasis; RVVC, recurrent vulvovaginal candidiasis.*

**TABLE 2:** Clinical and microbiological characteristics of patients included in this study.

Patient #	Age (years)	Species (MIC, μ g/ml)/ (isolate number)	Treatment	Final outcome

Patients with RVVC harboring resistant or high fluconazole MIC-responding isolates with/without prior short-term exposure to 1% topical clotrimazole (*n* = 5).				
29	29	*C. glabrata* (64)^A^	CLT 1% (12 days)	Persistent
		*C. glabrata* (64)	FLC 150 mg (first and fourth days = 2 doses)	
		*C. glabrata* (64)	FLC 150 mg (1 dose per week for 6 months = 24 doses)	
31	25	*C. krusei* (64)^A^	FLC 150 mg (2 doses)	Persistent
		*C. krusei* (64)	FLC 150 mg (2 doses)	
		*C. krusei* (64)	FLC 150 mg (24 doses)	
32	31	*C. krusei* (64)^A^	FLC 150 mg (2 doses)	Cleared
		*C. krusei* (64)	FLC 150 mg (24 doses)^B^	
34	31	*C. krusei* (64)^A^	Oral fluconazole 150 mg (first and fourth days)	Persistent
		*C. krusei* (64)	FLC 150 mg (2 doses) + CLT1% (10 days)	
		*C. krusei* (64)	FLC 150 mg (24 doses)	
41	41	*C. albicans* (0.125)	CLT 1% (12 days)	Cleared

**Patients with RVVC harboring fluconazole-non-susceptible isolates with prior long-term exposure to 1% topical clotrimazole (*n* = 7).**				

26	40	*C. albicans* (64)	FLC 150 mg (2 doses)	Cleared
60	27	*C. albicans* (16)	FLC 150 mg (2 doses)	Cleared
62	52	*C. albicans* (64)	FLC 150 mg (2 doses)	Cleared
63	38	*C. albicans* (64)	FLC 150 mg (2 doses)	Cleared
102	31	*C. albicans* (64)	FLC 150 mg (2 doses)	Cleared
118	29	*C. albicans* (4)	FLC 150 mg (2 doses)	Cleared
125	29	*C. albicans* (4)	FLC 150 mg (2 doses)	Cleared

**Patients with RVVC harboring fluconazole-tolerant isolates that became fluconazole-non-susceptible during the course of treatment (*n* = 3).**				

3	46	*C. albicans* (0.25)	CLT1% (12 days)	Persistent
		*C. albicans* (0.5)	FLC 150 mg (2 doses) + CLT 1% (10 days)	
		*C. albicans* (2) (tolerant)	FLC 150 mg (2 doses)	
		*C. albicans* (16)	FLC 150 mg (24 doses)	
4	35	*C. albicans* (0.25)	CLT 1% (12 days)	Persistent
		*C. albicans* (2) (tolerant)	FLC 150 mg (2 doses)	
		*C. albicans* (4)	FLC 150 mg (24 doses)	
20	20	*C. albicans* (0.25) (tolerant)^A^	FLC 150 mg (2 doses)	Persistent
		*C. albicans* (64)	FLC 150 mg (2 doses) + CLT 1% (10 days)	
		*C. albicans* (64)	FLC 150 mg (24 doses)	

**Patients with RVVC harboring tolerant isolates with/without prior short-term exposure to 1% topical clotrimazole (*n* = 8).**				

19	34	*C. albicans* (0.25)	FLC 150 mg (2 doses)	Persistent
		*C. albicans* (0.5)	FLC 150 mg (2 doses) + CLT 1% (10 days)	
		*C. albicans* (2) (tolerant)	FLC 150 mg (24 doses)	
43	24	*C. albicans* (0.5)	FLC 150 mg (2 doses)	Persistent
		*C. albicans* (0.5)	FLC 150 mg (2 doses) + CLT 1% (10 days)	
		*C. albicans* (2)^A^ (tolerant)	FLC 150 mg (24 doses)	
59	42	*C. albicans* (0.5)^A^ (tolerant)	CLT 1% (12 days)	Persistent
		*C. albicans* (1)^A^ (tolerant)	FLC 150 mg (2 doses)	
		*C. albicans* (0.125)^A^ (tolerant)	FLC 150 mg (24 doses)	
73	30	*C. albicans* (0.125)	CLT 1% (12 days)	Persistent
		*C. albicans* (0.125)	FLC 150 mg (2 doses)	
		*C. albicans* (0.125) (tolerant)	FLC 150 mg (24 doses)	
101	35	*C. albicans* (0.125)	FLC 150 mg (2 doses)	Persistent
		*C. albicans* (0.125)	FLC 150 mg (2 doses) + CLT 1% (10 days)	
		*C. albicans* (0.25) (tolerant)	FLC 150 mg (24 doses)	
110	40	*C. albicans* (0.25)	FLC 150 mg (2 doses) + CLT 1% (10 days)	Persistent
		*C. albicans* (0.25) (tolerant)	FLC 150 mg (2 doses) + CLT 1% (10 days)	
		*C. albicans* (0.5) (tolerant)	FLC 150 mg (24 doses)	
134	23	*C. albicans* (0.125)	FLC 150 mg (2 doses)	Persistent
		*C. albicans* (0.125)	FLC 150 mg (2 doses) + CLT 1% (10 days)	
		*C. albicans* (0.25) (tolerant)	FLC 150 mg (24 doses)	
138	68	*C. albicans* (0.25)	CLT 1% (12 days)	Persistent
		*C. albicans* (0.25)	FLC 150 mg (2 doses)	
		*C. albicans* (1) (tolerant)	FLC 150 mg (24 doses)	

**Patients with RVVC harboring fluconazole-tolerant isolates with prior long-term exposure to 1% topical clotrimazole (*n* = 2).**				

37	31	*C. albicans* (0.125)	FLC 150 mg (2 doses)	Cleared
89	44	*C. albicans* (2)	FLC 150 mg (2 doses)	Cleared

**Patients with RVVC harboring fluconazole susceptible (fluconazole-non-tolerant) isolates (*n* = 18).**				

NA	24–55	*C. albicans* (0.125–1) (*n* = 39)	FLC (2 doses)→ FLC (2 doses) + CLT 1% (10 days)→ FLC (24 doses) (*n* = 10) FLC (2 doses)→ FLC (2 doses) + CLT 1% (2 doses) (*n* = 1) CLT 1%(10 days)→ FLC (2 doses)→ FLC (24 doses) (*n* = 2) CLT 1% (10 days)→ FLC (2 doses) (*n* = 1)	Cleared (*n* = 4) Persistent (*n* = 10)
NA	29–35	*C. glabrata* (1–2) (*n* = 11)	CLT 1%(10 days)→ FLC (2 doses)→ FLC (24 doses) (*n* = 2) Oral fluconazole 150 mg (2 doses)→ Oral FLC (2 doses)→ FLC (2 doses) + CLT 1% (24 doses) (*n* = 1) CLT 1%(10 days)→ FLC (2 doses) (*n* = 1)	Persistent (*n* = 4)

**Patients with VVC without prior exposure to 1% topical clotrimazole successfully treated during the first course (*n* = 38).**				

29 patients	20–57	*C. albicans* (0.125–2) (*n* = 29)	FLC (2 doses) (*n* = 15) CLT 1% (12 days) (*n* = 14)	Cleared
3 patients	19–41	*C. glabrata* (1–16) (*n* = 3)	CLT 1% (12 days) (*n* = 2) FLC 150 mg (2 doses) (*n* = 1)	Cleared
2 patients	38, 63	*C. parapsilosis* (0.125) (*n* = 2)	CLT 1% (12 days) (*n* = 1) FLC 150 mg (2 doses) (*n* = 1)	Cleared
3 patients	29–52	*C. krusei* (4–64)^A^ (*n* = 3)	FLC 150 mg (2 doses) (*n* = 2) CLT 1% (12 days) (*n* = 1)	Cleared
1 patient	42	*C. tropicalis* (1) (*n* = 1)	FLC 150 mg (2 doses) (*n* = 1)	Cleared

*^*A*^These isolates were recovered from a patient who had previous short-term exposure to intravaginal topical 1% clotrimazole. ^*B*^These patients were only examined by a gynecologist and refused to provide swab samples during follow-up. FLZ: fluconazole; CLT: clotrimazole.*

Bacterial co-infection was observed in 15.8 and 30.2% of patients with VVC (6/38) and RVVC (13/43), respectively; it was completely resolved in the VVC group after antibiotic treatment but persisted in the RVVC group up to the second (7/13; 54%) and third (2/13; 15.4%) follow-up. Moreover, four new incidences of bacterial co-infection were detected at the second follow-up; these cases were successfully treated with antibiotics.

### *Candida* Species Distribution

**FIGURE 1:**
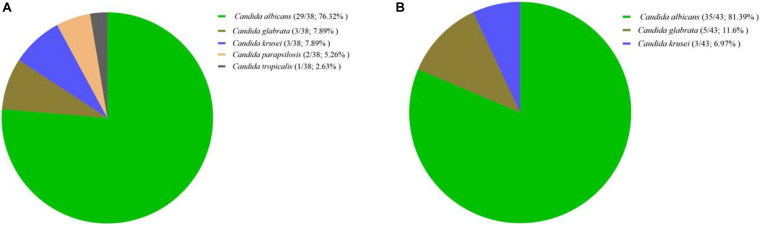
Distribution of *Candida* species isolates recovered from patients with vulvovaginal candidiasis **(A)** and recurrent vulvovaginal candidiasis **(B)**. Prevalence was noted per number of patients in each arm. Further details on isolate number are listed in Table 1.

In total, 145 yeast isolates were recovered from 43 patients with RVVC (107/145; 73.7%) and 38 patients with VVC (38/145; 26.2%); among them, *C*. *albicans* was the most prevalent species (114/145; 78.6%), followed by *C*. *glabrata* (17/145; 11.7%), *C*. *krusei* (11/145; 7.6%), *C*. *parapsilosis* (2/145; 1.3%), and *C*. *tropicalis* (1/145; 0.7%) (Tables 1, 2 and Figure 1).

The same trend was observed among the 81 patients: *C*. *albicans* was the most prevalent species in both VVC (29/38; 76.3%) and RVVC (35/43; 81.3%) groups (Table 1 and Figure 1). However, most of *C*. *glabrata* (14/17; 82.3%) and *C*. *krusei* (8/11; 72.2%) isolates were recovered from patients with RVVC (Table 1), whereas *C*. *parapsilosis* and *C*. *tropicalis* were only observed among those with VVC. All patients with RVVC carried the same species throughout the study period and mixed infections due to multiple yeast/*Candida* species were not observed.

### Association of FLZR and FLZT Phenotypes With Azole Therapeutic Failure

Resistance was noted only to fluconazole and only among patients with RVVC, whereas all *Candida* isolates recovered from patients with VVC were fluconazole-susceptible (Tables 2, 3). In total, 25.5% of RVVC cases (11/43) were due to fluconazole-non-susceptible (FNS) *C*. *albicans* (≥4 μg/ml) representing 15.3% of *C*. *albicans* isolates (13/85) recovered from the RVVC group (excluding cases due to mixed FLZR and FLZT isolates) (Tables 2, 3). Moreover, only one RVVC case was due to FLZR *C*. *glabrata* (MIC ≥ 64 μg/ml); this patient had a prior exposure to clotrimazole. Some of the FLZR isolates were obtained from patients who were repeatedly exposed to topical clotrimazole for a period of ≥1 year, but all the infections were cleared after two doses of fluconazole (patients # 26, 60, 62, 63, 102, 118, and 125) (Table 2). The other FLZR isolates emerged during the course of treatment with clotrimazole and/or fluconazole and both azoles showed therapeutic failure in the infected patients. Approximately 42% of patients with RVVC were infected with fluconazole-sensitive (FLZS) isolates, which were not tolerant to fluconazole (Table 2). On the other hand, all patients with VVC were successfully treated with either topical clotrimazole or oral fluconazole (Table 2). Although categorized as WT, 100 and 66.6% of *C*. *krusei* isolates recovered from patients with RVVC and VVC, respectively, responded to fluconazole MIC of 64 μg/ml. None of the isolates showed itraconazole resistance and 2.6% of *C. albicans* isolates showed susceptible dose-dependent phenotype (MICs 0.25–0.5 μg/ml) (Table 3).

**TABLE 3:** Species distribution and fluconazole and itraconazole susceptibility patterns of *Candida* isolates recovered from Iranian women suffering from VVC and RVVC.

Species	Susceptibility status	Fluconazole	Itraconazole
*C. albicans* (*n* = 114)	Susceptible	101	94
	Susceptible dose-dependent	3	20
	Resistant	10	0
	Range	0.125–64	0.032–0.25
*C. glabrata* (*n* = 17)	<ECV	14	17
	>ECV	3	0
	Susceptible dose-dependent	14	NA
	Resistant	3	NA
	Range	1–64	0.064–0.25
*C. krusei* (*n* = 11)	<ECV	11	11
	>ECV	0	0
	Range	4–64	0.032–0.25
*C. parapsilosis* (*n* = 2)	<ECV	2	2
	>ECV	0	0
	Susceptible	2	0
	Range	0.125	0.032
*C. tropicalis* (*n* = 1)	<ECV	1	1
	>ECV	0	0
	Susceptible	1	NA
	Range	NA	NA

*ECV, epidemiological cutoff value.*

The FLZT phenotype was only observed for *C*. *albicans* (13/114; 11.4%) and *C*. *tropicalis* (1/1; 100%) and was not detected among *C*. *glabrata*, *C*. *krusei*, and *C*. *parapsilosis* isolates. Interestingly, except for one *C*. *tropicalis* isolate from a patient with VVC who was successfully treated with two 150-mg doses of fluconazole, all FLZT *C*. *albicans* isolates were recovered from patients with RVVC (Table 2). The FLZT phenotype, similar to the FLZR phenotype, emerged either in the course of treatment or prior to the study, when the infected patients had long-term exposure to clotrimazole and already carried FLZT isolates before the recruitment to this study. Although two of the patients with prior clotrimazole exposure (# 37 and 89) were successfully treated with two doses of fluconazole, FLZT isolates which emerged during the course of treatment caused therapeutic failure of both fluconazole and clotrimazole (Table 2). Finally, FLZS isolates initially carried by some patients with RVVC acquired the FLZT and then the FLZR phenotype during the course of treatment, both of which were responsible for fluconazole and/or clotrimazole therapeutic failure.

## Discussion

Our study revealed that RVVC was dominant among the VVC cases, which, we believe, could be attributed to the disproportionate use of OTC topical clotrimazole, resulting in the emergence of FLZT and FLZR isolates. Moreover, we showed that, similar to the FLZR phenotype, the FLZT phenotype might predict azole therapeutic failure. Thus, our data confirm the multifactorial nature of RVVC, whose outcome can be influenced, along with host- and drug-related factors, by fungal characteristics such as FLZR and FLZT phenotypes.

Consistent with previous estimations (Denning et al., 2018), we showed that RVVC was a serious issue among Iranian women. Because VVC is considered a superficial and easy-to-treat fungal infection, most of the affected women refrain from visiting gynecologists and take OTC topical clotrimazole after consultation with pharmacists. Therefore, we assume that the prevalence of RVVC has been overestimated because of the low referral rate of patients with VVC, who have already been using topical clotrimazole. These findings highlight the importance of conducting nationwide observational prospective studies to define the actual prevalence of VVC and RVVC among Iranian women.

Our analysis of the yeast species causing vaginitis among Iranian patients revealed that *C*. *albicans* constituted 79% of the isolates and was the most abundant *Candida* species responsible for VVC and RVVC. This observation is consistent with previous studies on VVC (Sharifynia et al., 2017; Ghajari et al., 2018), oral candidiasis (Arastehfar et al., 2019a), and candidemia (Arastehfar et al., 2020d; Kord et al., 2020) in Iran, which documented the abundance of *C. albicans* among Iranian patients, whereas *C*. *glabrata* was reported the most prevalent *Candida* species among Indian patients (Mohanty et al., 2007). Of note, we observed that approximately 83% of *C*. *glabrata* and 73% of *C*. *krusei* isolates, which intrinsically respond to high MICs of azoles, were obtained from patients with RVVC. Altogether, this epidemiological profile indicates that *C*. *albicans* is the dominant yeast species in Iranian patients suffering from both VVC and RVVC; however, NAC species should also be a matter of concern.

Assessment of the azole susceptibility profiles of *Candida* isolates showed that *C*. *albicans* had the highest rate of the FLZR phenotype and was recovered only from patients with RVVC. This observation is in contrast with the typical situation in patients with candidemia, when *C*. *albicans* is rarely resistant to antifungal agents and fluconazole is the optimal treatment drug (Pfaller et al., 2019). Fluconazole resistance is most likely caused by repeated exposure to azoles, either fluconazole or OTC azoles. Similar to our findings, previous studies from Iran also reported a relatively high rate of fluconazole resistance among Iranian patients (Sharifynia et al., 2017). Since resistance to antifungal agents in general and to fluconazole in particular is associated with therapeutic failure, we assessed the clinical profiles of our patients, which showed that FLZR isolates mostly emerged after treatment with clotrimazole and some after that with fluconazole and were potentially associated with persistent RVVC. Bearing in mind that there is no ECV or CBP established for clotrimazole and that clotrimazole and fluconazole belong to various subset of azoles, imidazoles, and triazoles, respectively (Crowley and Gallagher, 2014), yet some clinical isolates with elevated MIC values against clotrimazole shown to be FLZR (Vazquez et al., 2001) and in some cases were cross-resistant to all azoles tested (Martel et al., 2010). Of note, none of our *Candida* isolates were itraconazole-resistant. Observing such heterogeneity in terms of resistance to a single or multiple azoles might be explained by underlying azole resistance mechanisms involved. For instance, some *Candida* isolates cross-resistant to multiple azoles harbor mutations in various *ERG* genes implicated in ergosterol biosynthetic pathway (Martel et al., 2010) in tandem with overexpression of efflux pumps, while some harboring a single mutation in *ERG11* may only confer resistance to fluconazole (Arastehfar et al., 2020a). Moreover, the structural differences noted among short-tailed, such as fluconazole, and long-tailed, such as itraconazole, may dictate the interaction with drug target followed by mutation type and hence the resistance to a single or multiple azoles, which may also vary depending on the species studied (Sagatova et al., 2015).

Along with drug resistance, drug tolerance has been revealed as an underestimated factor complicating patient treatment (Rosenberg et al., 2018; Arastehfar et al., 2020c; Berman and Krysan, 2020). Antifungal tolerance has not been evaluated in the context of VVC to clarify whether it is associated with azole exposure and can cause azole therapeutic failure. Our analysis of the antifungal tolerance rate and its correlation with the previous exposure to azoles and azole therapeutic failure showed that *C*. *albicans* was the only species developing tolerance against fluconazole among RVVC and that both short- and long-term exposure to azoles was associated with the development of tolerance. More importantly, FLZT isolates were likely associated with both fluconazole and clotrimazole therapeutic failure. Furthermore, in some cases *C*. *albicans* FLZT isolates showed an intermediate phenotype between FLZS and FLZR, further supporting the notion that drug tolerance paves the way for the emergence of drug resistance (Levin-Reisman et al., 2017; Windels et al., 2019). Indeed, studies in bacteria have indicated the importance of antibiotic tolerance and its contribution to higher resistance and therapeutic failure rates, as well as longer hospital stay and increased healthcare-related expenses (Levin-Reisman et al., 2017; Windels et al., 2019). Thus, antifungal tolerance is an emerging issue of significant clinical relevance, which necessitates the development of fast and straightforward techniques for accurate and rapid measurement of drug tolerance with the ultimate goal of improving patients’ outcomes.

Collectively, our and previous data indicate that FLZT and FLZR phenotypes may contribute to azole therapeutic failure in VVC treatment and that precautions, especially concerning the broad use of OTC topical azole preparations, should be taken. Moreover, *C*. *albicans* infections should not be regarded as easily treatable in the context of VVC, because the vast majority of persistent RVVC cases found in this study were caused by this species.

Another issue further complicating the management of VVC is the prescription of antifungal agents in the absence of species identification and AFST, which has also been revealed in a previous study of Iranian patients with candidemia (Arastehfar et al., 2020d), suggesting that VVC is an underestimated complication and a growing challenge for the healthcare in Iran.

Of note, approximately 42% of patients with RVVC recruited to this study were infected with azole susceptible non-tolerant isolates, but still showed persistent vaginitis despite treatment with fluconazole and/or clotrimazole, which is in line with the reports that host-related factors such as specific mutations causing immune deficiency and/or local immune overreaction may result in therapeutic failure (Jaeger et al., 2013; Costa-de-Oliveira and Rodrigues, 2020). This aspect will be the subject of our future studies, where we will try to categorize the most important mutations in the genes involved in host immunity. Furthermore, the fact that some patients with RVVC may not have adhered to the prescribed antifungal regimen could also explain a relatively high rate of therapeutic failure in the absence of fluconazole resistance or tolerance.

Since this was a single-center study, we admit that some VVC cases may have been missed, which is a limitation. We believe that a multicenter nationwide study would further the knowledge on the prevalence and severity of RVVC in Iran.

## Data Availability Statement

The original contributions presented in the study are included in the article/Supplementary Material, further inquiries can be directed to the corresponding author/s.

## Ethics Statement

The studies involving human participants were reviewed and approved by the human subject hospital review board of Shahid Akbar-Abadi Obstetrics and Gynecology Hospital (IR.MODARES.REC.1397.225). The patients/participants provided their written informed consent to participate in this study.

## Author Contributions

AA, MR, SM, WF, DP, and CL-F designed and coordinated the study. MK, NG, MR, and SM collected the isolates and clinical data. AA, FD, CL-F, MI, MT, MH, JJ, LH, HW, WF, and AC performed the species identification and antifungal susceptibility testing. AA drafted the manuscript and all authors revised the draft. WF and SM funded the study. All authors contributed to the article and approved the submitted version.

## Conflict of Interest

DP receives research support and/or serves on advisory boards for Amplyx, Cidara, Scynexis, N8 Medical, Merck, Regeneron, and Pfizer. The remaining authors declare that the research was conducted in the absence of any commercial or financial relationships that could be construed as a potential conflict of interest.
