# Supplementary material for: A High Rate of Recurrent Vulvovaginal Candidiasis and Therapeutic Failure of Azole Derivatives Among Iranian Women
Source: Front Microbiol. 2021 Apr 28;12:655069. doi: 10.3389/fmicb.2021.655069 (PMC8113757; doi:10.3389/fmicb.2021.655069)
Supplement: Supplementary file 1 [file Table_1.DOCX]

Supplementary table 1. List of covered species and associated primers for the first multiplex PCR

| **Species** | **Product size** | **Target gene** | **Primers** | **Sequence** | **Quantity (picomol)** | **PCR Conditions** |
| --- | --- | --- | --- | --- | --- | --- |
| ***C. albicans*** | **606 bps** | **IGS 1** | **Falb** | **AGATTATTGCCATGCCCTGAG** | **5** | 95 ℃, 5 minutes,  35 cycles (95 ℃, 30 seconds, 60 ℃, 30 seconds, 72 ℃, 30 seconds),  72 ℃, 8 minutes |
|  |  |  | **Ralb** | **CCATGTCGAACGTAGCGTAT*** | **5** |  |
| ***C. auris*** | **331 bps** | **26S rDNA** | **Faur** | **GAACGCACATTGCGCCTTGG** | **5** |  |
|  |  |  | **Raur** | **TCCAAAGGACTTGCCTGCT** | **5** |  |
| ***C. dubliniensis*** | **718 bps** | **IGS 1** | **Fdub** | **GTCGGACATATACCTCCAACTC** | **5** |  |
|  |  |  | **Rdub** | **CCATGTCGAACGTAGCGTAT*** | **5** |  |
| ***C. glabrata*** | **212 bps** | **hypothetical protein gene** | **Fgla** | **ACCGTGCTTGCCTCTACA** | **2** |  |
|  |  |  | **Rgla** | **GACATCTGAGCCTCGTCTGA** | **2** |  |
| ***C. parapsilosis*** | **490 bps** | **Phospholipase 1 gene (AJ320260.1)** | **Fpara** | **TACACCAAGCGACTCAGC** | **5** |  |
|  |  |  | **Rpara** | **ACCAGCTGCTTTGACTTG** | **5** |  |
| ***P. kudriavzevii*** | **1159 bps** | **Secreted Aspartic Proteinase gene** | **FKru** | **GGCGTTGTCCATCCAATG** | **5** |  |
|  |  |  | **Rkru** | **CAGGAGAATTGCTGTTCCC** | **5** |  |
| ***C. tropicalis*** | **126 bps** | **IGS 1** | **Ftro** | **AGAACAAGAAAACAGTGAAGCAA** | **5** |  |
|  |  |  | **Rtro** | **CCATGTCGAACGTAGCGTAT*** | **5** |  |

****Reverse primer shared by C. albicans and C. dubliniensis.***

Supplementary table 2. List of covered species and associated primers for the second multiplex PCR

| **Species** | **Product size** | **Target gene** | **Primers** | **Sequence** | **Quantity (picomol)** | **PCR Conditions** |
| --- | --- | --- | --- | --- | --- | --- |
| ***D. hansenii*** | **818 bps** | **ITS rDNA** | **Fam-F** | **GGATCTCTTGGTTCTCGCA*** | **5** | 95 ℃, 5 minutes,  35 cycles of (95 ℃, 30 seconds, 62 ℃, 30 seconds, 72 ℃, 30 seconds),  72 ℃, 8 minutes |
|  |  |  | **Fam-R** | **GCGAGGAACCCAACCAAGA** | **5** |  |
| ***M. guiliermondii*** | **302 bps** | **ITS rDNA** | **Guil-F** | **GGATCTCTTGGTTCTCGCA*** | **5** |  |
|  |  |  | **Guil-R** | **CCAGAAATATCCCGCCACA** | **5** |  |
| ***Kl. Marxianus*** | **203 bps** | **ITS rDNA** | **Kef-F** | **GGATCTCTTGGTTCTCGCA*** | **5** |  |
|  |  |  | **Kef-R** | **ACTTTCAAGTTAACCCGAGAC** | **5** |  |
| ***Y. lipolytica*** | **149 bps** | **RPB-2** | **Lipo-F** | **ACCGAGAGCGACGAGTA** | **5** |  |
|  |  |  | **Lipo-R** | **CTTTCTACCCAGAGCCACAA** | **2** |  |
| ***Cl. lusitaniae*** | **377 bps** | **ITS rDNA** | **Lusi-F** | **GGATCTCTTGGTTCTCGCA*** | **5** |  |
|  |  |  | **Lusi-R** | **CCGACTCAGACCACGAAAC** | **5** |  |
| ***P. norvegensis*** | **536 bps** | **RPB-2** | **Norve-F** | **GGGTTTGGAACCAATCTCAGA** | **5** |  |
|  |  |  | **Norve-R** | **GCAAAATCGGTGTTTTCGCTG** | **5** |  |
| ***D. rugosa*** | **689 bps** | **ITS rDNA** | **Rugo-F** | **GGATCTCTTGGTTCTCGCA*** | **5** |  |
|  |  |  | **Rugo-R** | **ACGGCCTTTTCACGAGAAGG** | **5** |  |

* Forward primers are shared *C. famata, C. Guiliermondii, C. kefyr, C. lusitaniae* and *C. rugosa.*

Supplementary table 3. List of covered species and associated primers for the third multiplex PCR

| **Species** | **Product size** | **Target gene** | **Primers** | **Sequence** | **Quantity (picomol)** | **PCR Conditions** |
| --- | --- | --- | --- | --- | --- | --- |
| ***Cryptococcus deneoformans*** | **235 bps** | **IGS** | **Deneo-F** | **CCATCCTGTTGGCGAAGA^*^** | **5** | 95 ℃, 5 minutes**,**  35 cycles of (95 ℃, 30 seconds, 60 ℃, 30 seconds, 72 ℃, 30 seconds)**,**  72 ℃, 8 minutes |
|  |  |  | **Deneo-R** | **GGTGCTGTATGAAGGCTATGG^**^** | **5** |  |
| ***Cryptococcus neoformans*** | **392 bps** | **IGS** | **Neo-F** | **CCATCCTGTTGGCGAAGA^*^** | **5** |  |
|  |  |  | **Neo-R** | **GGTGCTGTATGAAGGCTATGG^**^** | **5** |  |
| ***Cryptococcus gattii*** | **184 bps** | **IGS** | **Gattii-F** | **CAGGAGTGGATTCAGCGT** | **5** |  |
|  |  |  | **Gattii-R** | **GGTGCTGTATGAAGGCTATGG^**^** | **5** |  |
| ***Geotrichum candidum*** | **299 bps** | **18s rDNA** | **Gcandi-F** | **AGATTGTATCTTGAGAGCGGATTA** | **5** |  |
|  |  |  | **Gcandi-R** | **GCCGAAACACAGTTGAACAA** | **5** |  |
| ***Rhodotorula mucliginosa*** | **111 bps** | **18s rDNA** | **Rhodo-F** | **GCCTAGCTCGTTCGTAATGC** | **5** |  |
|  |  |  | **Rhodo-R** | **TTAACCCAACCCGGCTCT** | **5** |  |
| ***Trichosporon asahii*** | **483 bps** | **18s rDNA** | **Tasahii-F** | **GAAGGATCATTAGTGATTGCCTT** | **5** |  |
|  |  |  | **Tasahii-R** | **TACCTGATTTCAGGCCAGAG** | **5** |  |
| ***Trichosporon lactis*** | **480 bps** | **18s rDNA** | **Tlactis-F** | **GAAGGATCATTAGTGATTGCCTT** | **5** |  |
|  |  |  | **Tlactis-R** | **TACCTGATTTCAGGCCAGAG** |  |  |

* Forward primers are shared between *Cryptococcus deneoformans* and *Cryptococcus neoformans*

** Reverse primer is shared for *Cryptococcus deneoformans*, *Cryptococcus gattii*, and *Cryptococcus neoformans*
